# Ferroptosis and PPAR-gamma in the limelight of brain tumors and edema

**DOI:** 10.3389/fonc.2023.1176038

**Published:** 2023-07-24

**Authors:** Eduard Yakubov, Sebastian Schmid, Alexander Hammer, Daishi Chen, Jana Katharina Dahlmanns, Ivana Mitrovic, Luka Zurabashvili, Nicolai Savaskan, Hans-Herbert Steiner, Marc Dahlmanns

**Affiliations:** ^1^ Department of Neurosurgery, Paracelsus Medical University, Nuremberg, Germany; ^2^ Department of Trauma, Orthopaedics, Plastic and Hand Surgery, University Hospital Augsburg, Augsburg, Germany; ^3^ Center for Spine and Scoliosis Therapy, Malteser Waldkrankenhaus St. Marien, Erlangen, Germany; ^4^ Department of Otorhinolaryngology, Shenzhen People's Hospital, Jinan University, Shenzhen, China; ^5^ Institute for Physiology and Pathophysiology, Friedrich-Alexander-Universität (FAU) Erlangen-Nürnberg, Erlangen, Germany; ^6^ Department of Cardiac Surgery, Bogenhausen Hospital, Munich, Germany; ^7^ Department of Neurosurgery, Inova Medical Center, Tbilisi, Georgia; ^8^ Department of Neurosurgery, University Medical School Hospital Universitätsklinikum Erlangen (UKER), Friedrich-Alexander Universität (FAU) Erlangen-Nürnberg, Erlangen, Germany; ^9^ Department of Public Health Neukölln, District Office Neukölln of Berlin Neukölln, Berlin, Germany

**Keywords:** glioblastoma, glutamate, peritumoral edema, SLC7A11, ferroptosis

## Abstract

Human malignant brain tumors such as gliomas are devastating due to the induction of cerebral edema and neurodegeneration. A major contributor to glioma-induced neurodegeneration has been identified as glutamate. Glutamate promotes cell growth and proliferation in variety of tumor types. Intriguently, glutamate is also an excitatory neurotransmitter and evokes neuronal cell death at high concentrations. Even though glutamate signaling at the receptor and its downstream effectors has been extensively investigated at the molecular level, there has been little insight into how glutamate enters the tumor microenvironment and impacts on metabolic equilibration until recently. Surprisingly, the 12 transmembrane spanning tranporter xCT (SLC7A11) appeared to be a major player in this process, mediating glutamate secretion and ferroptosis. Also, PPARγ is associated with ferroptosis in neurodegeneration, thereby destroying neurons and causing brain swelling. Although these data are intriguing, tumor-associated edema has so far been quoted as of vasogenic origin. Hence, glutamate and PPARγ biology in the process of glioma-induced brain swelling is conceptually challenging. By inhibiting xCT transporter or AMPA receptors in vivo, brain swelling and peritumoral alterations can be mitigated. This review sheds light on the role of glutamate in brain tumors presenting the conceptual challenge that xCT disruption causes ferroptosis activation in malignant brain tumors. Thus, interfering with glutamate takes center stage in forming the basis of a metabolic equilibration approach.

## Introduction

Malignant primary brain tumors account for approximately 30% of all primary brain tumors diagnosed annually in the United States (1). Gliomas are the most common of these and represent one of the leading causes of morbidity and mortality in neurological practice (2). Glioblastomas (also referred to CNS WHO grade 4 glioma) with their median survival time of less than 15 months are considered to be the most malignant brain tumor entity (3). To date, conventional treatment includes surgical resection of the bulk tumor mass, followed by radiotherapy and alkylating agent-based chemotherapy. Even with these advanced therapies developed over the last two decades, survival times have only been extended a few months, and a cure remains elusive. In addition, certain biological properties of glioma make complete surgical resection nearly impossible and radiochemotherapy less effective or ineffective in treating residual glioma cells (4–6). At the cellular level, treatment resistance can be explained by the intra- and intertumoral heterogeneity observed in glioblastomas. Based on genomic and transcriptomic analyses of bulk tumors, glioblastomas can be categorized into four molecular subtypes, namely proneural, neural, classical, and mesenchymal (7, 8). However, a follow-up study revealed that all molecular subtypes coexist within a brain tumor heterogeneously (5). The clinical prognosis remained unaffected except for individuals belonging to the proneural subtype. In glioblastomas with high proportions of alternative subtypes, patients with dominant proneural subtype had poorer survival outcomes. In addition, the existence of glioblastoma stem cells (GSC) also contributes to resistance to adjuvant therapy and promote tumor recurrence (9).

Untreated cases of glioblastoma are commonly associated with perifocal edema resulting from blood-brain barrier disruption. These events can lead to devastating neurological sequelae, such as hemiparesis or cognitive decline (10). Whether the tumor-related edema zone should be resected presents a controversial issue until now, but according to a recent study, surgical resection of the peritumoral edema zone has been found not to carry a greater risk of postoperative complications. It even delayed tumor recurrence than simply removing the contrast-enhancing tumor alone (11).

There has been an association between glioblastoma-induced edema and alterations of tumor-associated genes inside the edema region, in terms of upregulations of e.g. c-myc, ERK, or AKT, and downregulation of tumor-suppressors such as p53 (11). Additional bioinformatic analysis of ‘The Cancer Genome Atlas’ (TCGA) data revealed that occurrence of tumor-related brain edema affects inflammatory gene expression, e.g. by increasing IL-10 levels (12), which in turn was shown to promote glioma cell invasion (13, 14). As a result, the threshold for T cell activation can be raised, and their antitumor activity can be directly suppressed (15). Aside from that, HMOX1-positive myeloid cells are also capable of secreting IL-10, causing T cell dysfunction and immune evasion (16). These recent data strongly suggest that these edema-enriched genes are crucial for gliomagenesis and tumor angiogenesis. The presence of these gene alterations in the edema region is in line with the observation that this area represents the biologically active part of the glioma microenvironment (6).

**Figure 1:**
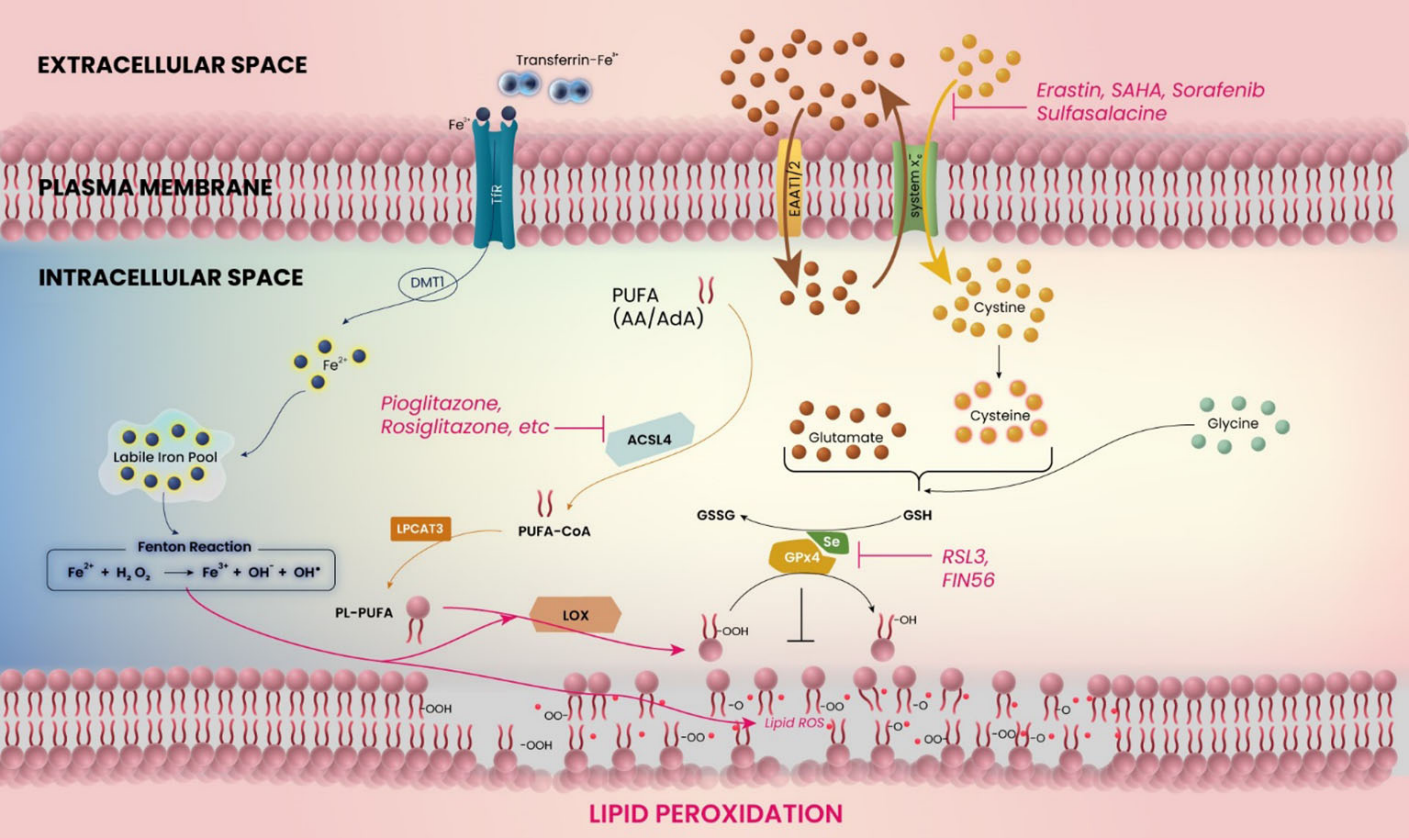
Schematic model for the mechanism of ferroptosis in glioblastoma. The figure shows the related molecules and pathways of ferroptosis. Ferroptosis is induced by inhibition of system x_c_^−^ or glutathione peroxidase 4 (GPX4), or accumulation of iron (Fe^2+^) ions. The catabolic enzyme acyl-CoA synthetase long-chain family member 4 (ACSL4) must be expressed.

Brain tissues in the peritumoral area show evidence of oxidative stress, manifested through increased production of reactive oxygen species (ROS) and decreased antioxidant-enzyme defenses such as catalase, glutathione peroxidase, and superoxide dismutase (17, 18). The oxidative stress in the glioma microenvironment is closely related to iron homeostasis, since the balanced amount of intracellular iron governs the oxidation state of phospolipids (19, 20) (**Figure 1**). An iron overload induces lipid peroxidation and subsequent cell death (21). In a recent study, it was found that GSC can absorb iron from the glioma microenvironment more effectively by upregulating their expression levels of ferritin and transferrin receptor (TfR) 1 (22). The proliferation of glioma cells is facilitated by such TfR overexpression-mediated oxidant accumulation, which inactivates cell cycle regulators and promotes S-phase entry (23).

In addition to the direct iron-related mechanism of tumor progression, another relevant upstream mechanism for the development of edema or glutamate-induced excitotoxicity is represented by the glutamate-cystine antiporter system x_c_^−^ (24, 25) (**Figure 1**). In glioblastoma cells, increasing cystine import through system x_c_^−^ drives the production of antioxidant glutathione, whereas the inhibition of cystine import, e.g. by application of system x_c_^−^ inhibitors, decreases the antioxidative capabilities of (brain) tumor cells (26). Several molecular aspects modulate these antioxidative properties, including the expression of mitoferrin-1, Nrf2, and catalase, among others (27–29). Thus, the tumor itself expresses a variety of molecules that serve to decrease the oxidative stress in its tissue, promoting growth. Interestingly, blocking system x_c_^−^ allows oxidative agents to accumulate intracellularly, which leads to cell death in the form of ferroptosis (30) (**Figure 1**). Ferroptosis inhibits malignant brain tumors and tumor-related edema by inducing oxidative stress in tumor cells and through antagonism of the treatment resistance that is strongly displayed by malignant gliomas (31).

In recent years, different pathways have been identified that promote an understanding of the biological actions of malignant tumors and their related cerebral edema. The benefits of current treatment options are still modest, so the investigation of newly identified, tumor- and edema-specific targets could translate quickly into clinical applications, ultimately improving survival rates and quality of life for patients. In this review, we outline recent advances in the treatment of tumor-associated cerebral edema and discuss the overlap between ferroptosis induction in the tumor and the role of ferroptosis adjacent to the edema site.

## Origins and relevance of tumor-induced cerebral edema

Aside from rapid growth and diffuse brain infiltration, peritumoral cerebral edema represents a feared hallmark of high-grade gliomas (HGGs, CNS WHO grades 3 and 4) (6, 32). This process involves an increase in fluid content in the surrounding parenchyma. As a result, the volume and, correspondingly, the clinical reflection of the mass-effect of the space-occupying intracranial process rises significantly (33, 34). According to their cause, there are four known types of cerebral edema, namely vasogenic, cytotoxic, interstitial, and osmotic edema (6, 32, 35–37). It cannot be denied that the vasogenic component is the major player in the progression of HGGs. In most cases, disruption of the blood-brain barrier and increased vascular permeability are responsible for the described fluid accumulation (38). This results in impaired oxygen transport, which increases the symptoms elicited by the edema (39). The disturbed fluid discharge increases intracranial pressure (40). This can be compensated in the first line by reduction of the intracerebral volume of blood and liquor (41), but soon this reserve is exhausted and the intracranial pressure rapidly rises. In the final stage, the swelling brain compresses essential brain areas as well as the venous outflow from the brain and thus results in an episode of ischemia that consequently leads to brain death. In fact, the malignancy of primary brain tumors correlates highly with the development of peritumoral edema (6, 17). The modified tumor microenvironment, known as perifocal or perilesional brain swelling, has been traditionally believed to originate primarily from vasogenic mechanisms and is also associated with the region of tumor-related angiogenesis (6, 42). Morphologically, tumor vessels show characteristic features such as altered capillary endothelial with fenestration of hyperplasia (glomeruloid tufts), irregular basal membranes and extravascular spaces, and also convoluted and sinusoidal abnormalities (43). As a consequence of this altered vascular architecture, gliomas accumulate extracellular water in the peritumoral zone while losing blood-brain barrier integrity and permeability selectivity (44). A crucial cellular component found within the tumor microenvironment is the astrocytic glial cell. Astroglial changes such as altered cytoskeletal arrangements, cytoplasmatic processes and filopodia, and altered expression of water channels (i.e. aquaporin-4) were indentified in perifocal areas (45, 46). These astroglial transformations may reflect a desperate attempt to restore the extracellular balance of fluids. In addition to astrocytes, microglia are also present in the tumor microenvironment and may influence the survival of patients (47). Recently, tumor-associated microglia/macrophages have been found to hold an important role in shaping the tumor microenvironment in mice (48). These data are particularly interesting because up to 50% of microglia/macrophages are estimated to constitute the tumor (49). Furthermore, beyond the bulk tumor mass, an analysis of the peritumoral zone has shown that activated microglia accumulate at the tumor and therby constitute a major component of the perifocal area that contributes to tumor-related edema (45). In total, the mechanisms of this perilesional edema and the cellular and molecular composition of the microenvironment are only partially understood, and further studies are required to assess how a cell-type specific intervention may elevate patients’ symptoms.

Based on the mostly vasogenic nature of peritumoral edema (44), it is relevant to identify the various angiogenic factors secreted by HGGs. The most prominent candidates associated with tumor angiogenesis are vascular endothelial growth factor (VEGF) and angiopoietin, as both can stimulate endothelial and perivascular progenitor cell growth as well as tube formation (50). Several mechanisms in which VEGF is involved, lead to an increase in the membrane permeability and thus are responsible for edema formation. Secretion of nitrogen oxide and phosphorylation of occludin are mainly driven by VEGF and result in relaxation of tight junctions (51). Another factor is the hydrostatic edema. This type of edema induces a shift in the liquor drainage due to the bulk tumor mass, creating a subsequent hydrostatic pressure gradient between the ventricle and brain parenchyma. As a result, fluid is forced into the brain tissue (52–54).

## Possibilities to broaden the therapeutic toolbox to treat perifocal edema

In clinical settings, the first choice remains the administration of synthetic glucocorticoids (i. e. dexamethasone) and, in rare cases, osmotically active compounds like mannitol (55). These clinical procedures usually reduce the edema rapidly. The underlying mechanisms consist of blocking nitrogen oxide synthase (NOS), accelerating the depletion of bradykinin, and reducing expression of VEGF by tumor cells (56, 57). These procedures stabilize the tight junctions and therefore reduce the efflux of capillary fluid into the brain parenchyma. However, there is also evidence that glucocorticoids may even accelerate the process of tumor progression (58). For instance, glucocorticoids have strong glycolytic effects and enhance fructose 2,6-bisphosphate production - the most potent stimulator of phosphofructokinase 1 - as well as lactate secretion, which may counteract the further action of anticancer drugs (59). Furthermore, frequent adverse reactions become increasingly relevant in long-term treatment with glucocorticoids, and can cause immuno-suppression, reduction in quality of life, and limiting treatment modalities (60).

With this wide variety of undesirable side effects, alternative treatment targets need to be indentified and utilized. Since it has been found that glutamate influx may contribute to cell-swelling (61, 62), therapeutic targeting of glutamate homeostastis-related proteins as system x_c_^-^ and EAAT1/2 may be potentially beneficial in treatment of tumor-related cerebral edema. In glioma, the decrease in EAAT2 (also known as solute carrier family 1 member 2 (SLC1A2) or glutamate transporter 1 (GLT-1)) correlates with tumor malignancy (63), making the potential involvement of EAAT in tumor-associated diseases much more relevant. For breast and colon cancers beneficial effects of EAAT2 upregulation have already been reported, and the antineoplastic effects have already been well studied (64, 65). Contrary to the system x_c_^-^, EAAT2 is poorly expressed by glioblastoma cells (66, 67). It regulates the entry of glutamate into these cells (**Figure 1**), ultimately decreasing extracellular clearance. Under normal conditions, EAAT2 is predominantly expressed in astrocytes, although detection is also possible in oligodendrocytes and neurons (68, 69). EAATs, in general, are membrane-bound pumps. Up to 90% of extracellular glutamate uptake can be accounted for by these transporters (70, 71), making them the single most important mechanism for a glutamate equilibrium. Upregulation of these receptors, in turn, leads to a substantial shift of extracellular to intracellular glutamate. Their expression can be modified by multiple substances and levels, including signaling pathways through PI3K and NF-κB, as well as EGF, PPARγ and pituitary adenylate cyclase-activating polypeptide (67, 71, 72).

When EAATs get activated, the sodium ion-driven uptake of glutamate also leads to the uptake of water, which may contribute to cytotoxic edema (54). However, observations from liver failure-induced cerebral edema suggest a decrease of EAAT2 accompanied by a concurrent increase in AQP4 expression (73, 74). These actions can elevate extracellular glutamate levels, until reaching neurotoxic amounts of glutamate leading to the activation of NMDA receptors associated with neurotoxicity (75, 76). Mechanistically, in terms of tumor-related edema, its formation can result from a rise in glutamate, as well as leukotrienes and vascular endothelial growth factors, which increase the permeability of the brain vessels surrounding the tumor, that leads to the influx of protein-rich influx in the brains’ white matter (77). In cases of the other forms of edema such as cellular or interstitial edema, the pathophysiological mechanism differs and may involve other consequences, e.g. increased sodium influx.

When AMPA receptor involvement in cerebral edema, tumor unrelated and evoked e.g. by traumatic brain injury, was assessed, it was found that blocking AMPA receptor activity attenuates edema (78–80). Interestingly, in studies using rodent ischemic models, AMPA-R and NMDA-R antagonistic actions have demonstrated promising results. However, despite these positive preclinical findings, clinical trials using AMPA receptor and NMDA receptor antagonists have been unsuccessful (81, 82). Nonetheless, the neuronal microenvironment near the tumor plays an important role for the tumor progression. In breast and prostate carcinoma cells, it was shown that tumor behavior is responsive to modulation of neurotransmitter activity (83), indicating the importance of chemical released by neuronal tissue. In the specific case of brain cancer, it is important to understand how malignant tumor cells and the neuronal cells in the brain communicate with each other in a reciprocal manner. Previous efforts have been made to address these questions. A soluble form of neuroligin-3, a synaptic protein, was able to activate PI3K-mTOR in high-grade glioma, and increased neuroligin-3 expression was negatively associated with patient survival (84). In addition to the dependency on such molecules, it was shown that glioma membrane depolarization drove tumor proliferation (85). In the same study, it was found that glioma provide electrical feedback to neurons in the circuit, thereby regulating their own, activity-driven growth. These data strongly illustrate how the neuronal compartment in the brain in the near vicinity of the tumor is involved in the tumor’s progression.

## xCT, microenvironment and tumor-associated cytotoxicity

As another glutamate level-regulating protein, the amino acid transporter xCT (system x_c_^-^, SLC7A11) is expressed in various cancers including high-grade gliomas (HGGs). Its specific modulation of tumor microenvironment is revealed to be a hallmark of primary malignant brain tumors. In particular, this modulation influences the tumor-induced neurotoxicity and perifocal edema (32). De- and methylation processes as well as imbalances between the histone deacetylases and acetylases play a critical role in tumor development, whereas the link between these epigenetic regulatory mechanisms and the malignant glioma progression is the transporter system (86).

The inhibition of the cystine/glutamate antiporter xCT leads to a decrease in neurodegeneration, perifocal edema and prolonged survival *in vivo*. Furthermore, this supports the hypothesis that the formation of edema is, to some extent, influenced by the death of peritumoral cells (24) (**Figure 1**). Inhibition of xCT primarily disrupts its neurodegenerative and microenvironment-toxifying activity (87). A decrease in glioma cell proliferation was associated with higher concentrations of xCT inhibitors in an L-cystine-dependent manner (24, 88, 89). Glioblastoma cells derived from human patients have been shown to be susceptible to the cytotoxic effects of xCT-inhibitors (90). Additionally, cytotoxic effects of substances such as temozolomide are augmented during sulfasalazine-mediated xCT inhibition (91), and in another study it was found that the glioma-toxic effect of sulfasalazine alone was detectable at concentrations above 200 µM (89). HGGs use xCT to increase glutamate levels and manipulate neuronal glutamate signaling for their own growth advantage, leading to chemotherapeutic resistance and a toxic tumor microenvironment for neurons. Reactive oxygen species (ROS) activate transient receptor potential (TRP) channels with the result of a potentiated glutamate release *via* the TRP-channels. The system x_c_^-^ modulates the tumor microenvironment with impact on host cells and the cancer stem cells (92).

Glioma-associated microglia/macrophages (GAMs) are an important cell population component of glioblastoma microenvironment. The increasing glutamate levels cause transcriptional changes in GAMs. These cells respond to extracellular glutamate excess in the glioblastoma microenvironment with increasing expressions of genes related to glutamate transport and metabolism such as GRIA2 (GluA2 or AMPA receptor 2), SLC1A2 (EAAT2), SLC1A3 (EAAT1), decreasing expression of xCT and increasing expression of GLUL (glutamine synthetase) (93).

Regarding the modulation of chemotherapeutic therapy of HGGs, many promising inhibitors and activators of xCT have been detected so far. The key problem of a specific modulation of xCT in gliomas is the ubiquitous expression of xCT also in vital tissue cells which makes it difficult to specifically target expression of xCT in tumor cells, especially because of its essential role in physiology of the CNS (94, 95).

The main cytotoxic tool in countering HGGs is the autophagy-inducing standard chemotherapeutic agent temozolomide. Interestingly, silencing xCT expression in human glioma cells is associated with a higher vulnerability towards temozolomide. However, gliomas with a high xCT expression are more vulnerable towards combinatorial treatment with temozolomide and erastin, a ferroptosis inducing agent (87).

The HDAC-inhibitor SAHA achieves equilibrium with the xCT transporter and is specific to malignant brain tumors, while leaving physiological xCT levels in healthy brain parenchyma unaffected. Consequently, the reduction of extracellular glutamate levels leads to a decrease in neuronal cell death and normalization of the tumor microenvironment. Reducing neurodegeneration results in less damage to the surrounding healthy brain parenchyma (94).

Activating transcription factor 4 (ATF4) is a critical oxido-metabolic regulator that contributes to the malignancy of HGGs by promoting cell proliferation, migration and tumor angiogenesis through the modification of the microenvironment in a potentially harmful way. ATF4 activation is associated with an elevation of xCT levels. The ATF4-induced proliferation is extenuated by xCT inhibition and ferroptosis inducers such as sorafenib and erastin. Moreover, erastin is able to reduce the ATF4-induced angiogenesis. ATF4 and xCT are tightly connected *via* a xCT-dependent configuring of the vascular architecture (31). Interestingly, ATF4 suppression comes along with an increased temozolomide susceptibility and autophagy in HGGs leading to a migratory stop after temozolomide application. ATF4 activation comes along with a xCT elevation resulting in an elevated temozolomide resistance. Thus, ATF4 can be regarded as a chemo-resistance gene in gliomas being determined by its transcriptional target xCT. Inactivation of ATF4 might be a key strategy to eliminate chemo-resistance in human gliomas (96). These findings open the door to new strategies of pharmacological interventions on tumor-associated genes by epigenetic priming (86).

## Involvment of ferroptosis in brain tumor treatment and the implications for associated edema

Ferroptosis is a recently discovered mechanism for cell death, characterized by the accumulation of iron ions and lipid peroxidation during cell death (30, 97) (**Figure 1**). This can ultimately be caused by ROS accumulation through inhibition of glutathione (GSH) or GSH-dependent selenoprotein glutathione peroxidase 4 (GPx4) (6, 30), with the latter being expressed most abundantly in testes and brains (98). This accumulation of iron ions can then lead to a continuous cycle of lipid oxidation and further iron accumulation. Interestingly, inhibitors of apoptosis, necrosis and autophagy cannot reverse this type of cell death (30).

Blocking system x_c_^-^, which displays a strong expression in malignant tumors such as glioblastoma, induces ferroptosis in the tumor cells. As this poses the question if system x_c_^-^ may be a potential target during chemotherapeutic intervention, it also of particular interest to examine whether ferroptosis is involved in the tumor-associated edema. Recently, it was found that the standard treatment for edema, dexamethasone, sensitizes cells to ferroptosis (99), which would allow to target the tumor and its edema simulatenously by potential medication regimens. *In vivo* treatment with the ferroptosis inducer sulfasalazine in mice with glioma also reduces the tumor-associated edema (89). In line with this finding, system x_c_^-^ inhibition by RNA-mediated silencing improves tumor associated-edema in glioma (24). In contrast to this improvement, rats after subarachnoid hemorrhage developed edema that improved after the inhibition of ferroptosis (100), instead of its induction. Thus, the underlying pathology that leads to edema seems to be important for the interventions to be taken in edema, and it may be possible that tumor-related edema appear to be more reliant on the ferroptosis status at the tumor site.

Therefore, it would be valuable to assess other ferroptosis-inducing drugs regarding their ability to modify tumor-related edema. At the moment, four distinct classes of ferroptosis-inducing drugs play a major role. Most important in treatment are class 1 and class 2 inducers (95, 101). However, class 3 (GSH depletion compounds, e. g. acetaminophen) and class 4 (lipid peroxidation inducers, e. g. FINO_2_) also play significant roles.

Class 1 inducers, such as erastin, primarily target the aforementioned transporter system x_c_^-^, ultimately depleting the cells of cystine and glutathione. Interestingly, it has been reported, that especially some glioma cells, that were therapy-resistent to current treatments, are characterized by increased synthesis of polyunsaturated fats (102) – a dependency that can be readily exploited by GPx4 inhibitors (103).

Class 2 inducers, such as Ras-selective lethal 3 (RSL3) and ferroptosis inducer 56 (FIN56), directly target and inhibit GPx4 through/via downstream process (101). This presents another therapeutic angle, as some trials with knockout human cancer cells have shown a partial resistance to erastin, but not RSL3 (104).

However, serious side effects have been reported involving the induction of ferroptosis in cardiomyocytes (105, 106). A probable explanation for this could be the counteraction of the vital, protective, and antioxidant role that GPx4 plays in many cell types (17, 107). Therefore, therapeutic use should be exercised with caution.

As a side note, a number of compounds can protect against ferroptosis-induced tissue damage. These include thiazolidinediones (TZDs), which are a class of PPARγ agonists (108, 109), as well as LOX-inhibitors, DPP4-mediated lipid peroxidation suppression and iron chelators (97).

Ultimately, ferroptosis-inducing agents such as erastin and RSL3 could potentially serve as extension to standard treatments, especially in cancers that seem resistant to current drug regiments. Potential side effects, however, should be taken into consideration.

## Role of PPARγ in brain tumors and cerebral edema

Glioma cells are represented by various cellular and molecular alterations that may contribute to their pathological effects and may also represent therapeutical targets. Amongst those altered signaling pathways, glioma cells express lower endogenous levels of peroxisome proliferator-activated receptor gamma (PPARγ) compared to healthy brain tissue (67, 110). PPARγ is a ligand-activated transcription factor that plays an important role in differentiation at a cellular level, as well as glucose and lipid homeostasis. It has been shown to inhibit cellular proliferation and angiogenesis, while promoting differentiation and inducing apoptosis through multiple pathways (111). One of the main obstacles for drugs designated for intracranial effect is the crossing of the blood-brain barrier. This can be easily overcome by PPARγ agonists, as demonstrated for pioglitazone in human glioma xenograft model (112). As shown in **Table 1**, a wide variety of antineoplastic efficacy could be seen with PPARγ agonists.

**Table 1:** Assessment of PPAR gamma agonists for their oncological value.

Author	Year	Agonist	Cells	Mechanism induced	Mechanism hindered
Grommes et al.	2013	Pioglitazone	LN-229		Tumor volume
Pestereva et al.	2012	Ciglitazone	T98G neurospheres, primary GSC	NANOG	SOX2
Wang et al.	2012	Rosiglitazone	U87MG, U251 MG		TGF-ß, P-SMAD3, SMAD3/SMAD4 complex
Wan et al.	2011	Pioglitazone	U87MG, U251 MG, T98G		ß-catenin
Lee et al.	2011	Pioglitazone	T98G		AKT, MMP
Charawe et al.	2008	Ciglitazone	U87, T98G neurospheres		EGF, Tyk2-STAT3
Coras et al.	2007	Troglitazone	SMA-560, U87MG, F98		TGF-ß, migration (organotopic model)
Spagnolo et al.	2007	Pioglitazone	GL261	Superoxide in glioma cells	
Grommes et al.	2006	Pioglitazone	C6, A172, U87MG		Ki67, tumour volume
Grommes et al.	2005	GW7845	C6, A172, U87MG		Ki67, tumour migration

While some effects in cerebral neoplasms and edema have been reported, the exact relation is not yet fully understood. To illustrate, rosiglitazone has been shown to cause G2/M arrest and apoptosis in certain glioblastoma cell lines (113), furthermore a delay in the age of onset of seizures has been demonstrated in genetically susceptible mice, when utilizing pioglitazone (114). In a clinical trial, an extended median survival of 19 months has also been reported for diabetic patients with glioblastoma who received additional treatment with PPARγ agonists, compared to 6 months of extended survival for patients receiving the standard treatment (115). However, it should be noted that the observed result indicating longer survival for the PPARγ agonist group was not considered statistically significant due to the small sample size used in the study. Currently, classic PPARγ agonists such as pioglitazone are FDA approved primarily as oral antidiabetics but the possibility of generating tissue-specific drugs has been validated (116). At the moment, treatment with first-generation TZDs poses many obstacles, as they hold a wide variety of side effects, limiting their use.

Recent studies have linked PPARγ to ferroptosis. For instance, dendritic cells in the immune system were shown to require PPARγ to undergo ferroptosis in response to RSL3, a finding that has an impact on antitumor immunity (117). The impact of PPARγ is not limited to cancer, since also other pathologies such as diabetic retinopathy were shown to be influenced in their ferroptotic behavior by PPARγ (118). In neuronal tissue, PPARγ-mediated ferroptosis was found to be relevant in the context of traumatic brain injury (119) and in intracerebral hemorrhage (120).

In addition to PPARγ’s role in tumor tissue and ferroptosis that ocurrs within, its importance also expands to edema. For example, in the context of traumatic brain injury PPARγ-modulating substances like pioglitazone and rosiglitazone led to decreases in edema in rodent models (121). Their use is, however, associated with peripheral edema (122). Though rosiglitazone, for example, has been shown to decrease edema following a hemorrhagic event (123), further studies are required to investigate PPARγ specifically in the context of cerebral edema as a result of an adjacent glioma.

According to studies in glioma cell lines and glioma stem cells, PPARγ agonist pioglitazone enhances the functional expression of EAAT2 (124). It suggests that glioblastoma cells at the peritumoral zone may be able to improve glutamate transport, which may lead to alleviation of tumor-related edema. PPARγ agonists have additional effects associated with lipid metabolism and ferroptosis (**Figure 1**). Polyunsaturated fatty acids (PUFAs) play a crucial role in the process of ferroptosis. To induce lipid peroxidation, the PUFA catabolic enzyme acyl-CoA synthetase long-chain family member 4 (ACSL4) must be expressed (109). This enzyme is essential for ferroptosis and responsible for esterifying CoA into PUFAs such as arachidonic acid (AA) and adrenic acid (AdA). By forming Acyl-CoA, PUFAs are activated for fatty acid oxidation. Utilizing pharmaceutical inhibition of ACSL4 with thiazolidinedione ligands, a class of PPARγ agonists such as pioglitazone and rosiglitazone, has demonstrated that PPARγ agonists can suppress ferroptosis sensitivity (109). Based on our current understanding, there is a notable absence of comprehensive and in-depth studies exploring the molecular mechanisms underlying the regulation of PPARγ and ferroptosis in glioblastoma. Further investigations on the role of PPARγ in glioma microenvironment and ferroptosis are required.

## Conclusion

In this review, we propose a fresh view on metabolic homeostasis in context of glioma-induced neurodegeneration and peritumoral edema. We discussed the glutamate signaling cascade and glutamate-EAAT-xCT axis with a special focus on ferroptosis. Brain tumors display a deranged microenvironment with metabolic changes. We discussed xCT and PPARγ as therapeutic targets addressing brain swelling and metabolic homeostasis. We provide supporting evidence for the conceptual challenge that xCT disruption causes ferroptosis activation in malignant brain tumors. We raised the potential involvement of PPARγ agonists in the context of glioblastoma and tumor-related edema.

## Author contributions

EY and MD designed the concept, structure and content of the review. EY and MD wrote the manuscript with input from SS, AH, DC, JKD, IM, LZ, H-HS, and NS. EY, SS and MD prepared all tables and figures. All authors listed provided critical revisions to the article. All authors contributed to the article and agreed to submit the manuscript in its current state.
